# Geospatial and Temporal Analysis of Avian Influenza Risk in Thailand: A GIS-Based Multi-Criteria Decision Analysis Approach for Enhanced Surveillance and Control

**DOI:** 10.1155/2024/6474182

**Published:** 2024-09-13

**Authors:** Waratida Sangrat, Weerapong Thanapongtharm, Suwicha Kasemsuwan, Visanu Boonyawiwat, Somchai Sajapitak, Chaithep Poolkhet

**Affiliations:** ^1^ Bureau of Disease Control and Veterinary Services Department of Livestock Development, Bangkok 10400, Thailand; ^2^ Faculty of Veterinary Medicine Kasetsart University, Kamphaeng Saen, Nakhon Pathom 73140, Thailand; ^3^ Akkhraratchakumari Veterinary College Walailak University, Thasala, Nakhon Si Thammarat 80161, Thailand

**Keywords:** highly pathogenic avian influenza, risk, spatial, temporal, Thailand

## Abstract

Avian influenza (AI) is a viral infection that profoundly affects global poultry production. This study aimed to identify the spatial and temporal factors associated with AI in Thailand, using a geographic information system (GIS)–based multi-criteria decision analysis (MCDA) approach. We discovered that high-risk areas for AI were primarily concentrated in the central and lower northern regions of the country, with fewer occurrences in the northeastern and southern regions. Model validation using historical outbreak data showed moderate agreement (AUC = 0.60, 95% CI = 0.58–0.61). This study provides valuable insights for planning national AI surveillance programs and aiding in disease prevention and control efforts. The efficiency and effectiveness of disease surveillance at the national level can be improved using this GIS-based MCDA, in conjunction with temporal risk factor analysis.

## 1. Introduction

Avian influenza (AI) is an important poultry viral disease that has a significant impact on the global poultry production industry. In Thailand, there have been no reports of the incidence of AI since 2008; therefore, regular implementation and improvement of effective disease surveillance systems is required. For optimum performance, surveillance systems should be able to detect the circulation of both highly pathogenic avian influenza (HPAI) and low pathogenic avian influenza (LPAI), to facilitate detection of outbreaks in early stages. The European Commission also indicated that risk-based surveillance methods, which include temporal considerations, should be used for disease surveillance [1].

Several risk factors for AI—such as proximity to other poultry farms, surface water, unprotected waterfowl habitats, presence of wild birds, backyard production systems, free-grazing ducks, commercial poultry farms, high poultry farm density, trade-related transport, and bird movements—have been identified by previous studies [2–11]. Farms with undetected clinical signs of viral circulation may still experience LPAI. Possible factors associated with LPAI include some specific avian species, poultry flocks having variations in age and species of poultry, old-aged poultry birds, and insufficient biosecurity measures [1, 12–14]. Wild waterbirds, such as waterfowls, gulls, terns, shorebirds, storks, cranes, and rails, are natural reservoirs of AI virus; traveling of these birds across continents can infect domestic poultry and other birds [10]. Studying factors associated with AI is also important for acquiring knowledge that can help detect temporal risks and can be used in surveillance systems [1, 15]. Temporal factors associated with AI in Korea have been reported by a previous study. The researchers found that variation in monthly habitat suitability of waterfowl significantly contributed to the prediction of risk of disease occurrence [16]. In addition, the timing of H5N1 outbreaks in Asia is closely related to the movement of poultry and migrating birds [17, 18]. Several studies have used data from previous outbreaks to identify seasonal patterns of HPAI [19, 20].

Geographic information system (GIS)–based multi-criteria decision analysis (MCDA) is a useful tool for identifying at-risk areas and supporting decision-making systems [21–23]. Therefore, using GIS-based MCDA in conjunction with temporal risk factor analysis will aid in planning AI surveillance at a national level. In this study, we aimed to identify possible spatial and temporal factors associated with AI in Thailand, using GIS-based MCDA. The results of this study indicate a need for prioritization of national surveillance programs to prevent and control disease occurrence and spread.

## 2. Methodology

### 2.1. Study Framework

A GIS-based MCDA was used to identify the possible spatial and temporal risk factors for AI in Thailand. This study collected data from May 2022 to February 2023. A literature review was conducted on articles published between 2007 and 2022, using PubMed (https://pubmed.ncbi.nlm.nih.gov/), Google Scholar (https://scholar.google.com/), and Scopus (https://www.scopus.com/). This included articles on poultry movements, observations of wild birds, outbreaks of AI associated with migratory flyways, proximity to outbreaks in neighboring countries, proximity to colonial water birds and water bodies, water bird density, backyard poultry density, free-grazing duck density, farm duck density, and poultry holding density. Subsequently, four experts, one epidemiologist and three veterinary officers who worked on and were experienced with HPAI outbreaks and controls, were contacted via e-mail and asked to add possible spatial and temporal risk factors to the list. By consensus, the experts added the following risk factors: temporal effects on introduction and/or disease spread, number of outbreaks of AI in Thailand, layer density, broiler density, and proximity to AI ELISA-positive farms. Experts were also asked to define the relative importance of each factor using a pairwise comparison technique.

Data from the basic eBird dataset (from 2000 to 2021) was obtained from eBird (https://ebird.org/home) [24]; this dataset contains the number of migratory and local birds based on the observation dates, reported by voluntary bird watchers. Therefore, the data relies on the density and probability of wild birds being observed. Data from the Department of Livestock Development (DLD) focused on colonial waterbirds, backyard poultry density, free-grazing duck density, poultry movement data, poultry population, poultry holding, and AI ELISA-positive farms, covering the period from January to December 2021. Additionally, the DLD provided data pertaining to HPAI outbreak in Thailand, from 2004 to 2008. Data from the Department of Provincial Administration (DOPA) contained information on administrative boundaries in Thailand. Furthermore, data from the World Organization for Animal Health (WOAH) [25] focused on the number of HPAI outbreaks that occurred between May 2021 and April 2022; the United Nations Office for the Coordination of Humanitarian Affairs (OCHA) [26] provided data on inland water bodies in Thailand. These data sources were used to assess the spatial and temporal risks.

In this study, we focused on both HPAI and LPAI in our data analysis, collectively referred to as AI. However, there is only data available on the number of outbreaks of HPAI in countries within the East Asian–Australasian Flyway (EAAF). This data specifically pertains to HPAI. This approach ensures a comprehensive risk assessment and supports the development of effective surveillance strategies.

Written informed consent was obtained from all experts regarding ethical issues using Google Forms. All the procedures were checked in accordance with the principles of the Declaration of Helsinki.

### 2.2. Raw Data of Spatial and Temporal Risk

All spatial risk factors listed from the literature review, expert opinions, and empirical data were divided into two categories: (1) risk of introducing AI from neighboring countries to the poultry population at risk in Thailand, and (2) risk of spread of AI throughout Thailand. From this list of possible risk factors, the top six most important factors in each category were selected by experts (Supplementary 1). In terms of disease introduction, the identified spatial risk factors included: proximity to outbreaks in neighboring countries, colonial waterbirds, water bodies, water bird density, backyard poultry density, and free-grazing duck density. The selected spatial risk factors for disease spread were: live poultry movement, layer density, broiler density, farm duck density, poultry holding density, and proximity to AI ELISA-positive farms. Then, using a pairwise comparison test with a nine-point continuous scale, each factor was compared to the other factors in each category, and each component was weighted according to the degree of its significance (Supplementary 2). The steps for this method are summarized in a flowchart (Supplementary 3).

Five important temporal risk factors were selected by the experts: temporal effects on introduction and/or disease spreads; number of poultry (broiler/chicken breeder/layer/laying duck/meat type duck/native chicken) movement at the provincial level by month, including the purpose of movement (rearing/others); number of targeted bird species (orders: Accipitriformes, Anseriformes, Charadriiformes, Ciconiiformes, Gruiformes, and Pelecaniformes) by month; number of outbreaks of HPAI in countries within the EAAF by month; and the number of outbreaks of HPAI in Thailand during 2004–2008, as shown in Supplementary 1. The experts also compared the importance of each factor to that of the others using a nine-point continuous scale (Supplementary 2). Subsequently, we weighed each factor to determine its importance. Then, the standardized data for each factor were multiplied by their weights and combined to identify temporal risk (Supplementary 1 and 3).

### 2.3. The Experts and Pairwise Comparison

For pairwise comparisons using a nine-point continuous scale, all experts were asked to assign their scores to the intensities of importance. The weight value for each factor (*w*_*i*_) was calculated by taking the eigenvector corresponding to the largest eigenvalue of the pairwise score matrix, and then normalizing the sum of the components to unity. The consistency of the matrix was verified using consistency ratio, CR, which was calculated as the consistency index, CI, divided by random index, RI. For CI, it was calculated as follows:

(1) $$\begin{array}{c} \text{CI}=\frac{\lambda_{\max}-n}{n-1}, \end{array}$$

where *λ*_max_ is the maximum eigenvalue of the judgement matrix and *n* is the number of factors. RI is derived from Saaty [27] and is entirely dependent on the number of factors in the analysis (Supplementary 3). If CR is higher than 0.10, then some pairwise values need to be reconsidered and the process is repeated until the desired value of CR less than 0.10 is reached [27].

### 2.4. Data Transformation and Visualization

For spatial risk factors, data on the population density of backyard poultry, free-grazing ducks, layers, broilers, and farm ducks were calculated as the number of heads/km^2^ in a subdistrict and then converted to map layers. The poultry holding density was calculated as the number of farms/km^2^ at the subdistrict level, which was also transformed into a map layer. To limit the complex process of computerization, eBird data from 2017 to 2021 were used to calculate the number of focused water birds as the number of heads/km^2^ at the subdistrict level and were converted to a map layer. Proximities from each subdistrict to an outbreak in a neighboring country, colonial waterbirds, water bodies, and AI ELISA-positive farms were calculated using geographic coordinates and transformed into a map layer in kilometers (km) using the Euclidean distance. Live poultry movement at the subdistrict level, expressed as the number of movements into subdistricts (time/year), was transformed into a map layer. In this study, all geographical data and mapping were developed using ArcMap 10.2 (ESRI, Redlands, California, USA).

For the temporal risk data, poultry movement data using the number of movements were analyzed and displayed by species using a bar chart. The number of birds that were susceptible to AI, obtained from the eBird data during 2000–2021, was calculated and displayed as a number of birds in each month of each province using a bar chart. For the outbreak data, the monthly distribution of HPAI in countries located in the EAAF zone was created and shown using mapping and bar charts. In addition, we used the number of HPAI outbreaks that occurred in Thailand between 2004 and 2008 to visualize the seasonal patterns of AI occurrence. The outbreak data were then aggregated according to the number of outbreaks each month.

### 2.5. Data Standardization

Similar methods are used to determine the importance of spatial and temporal risk factors. However, standardizing and combining processes uses different approaches for spatial and temporal risk factors. The spatial risk factors were standardized using fuzzy membership functions. The standardized values of each factor layer and their weights were combined using a weighted linear combination (WLC) to generate a suitability map for further analysis. To standardize all layers to the common data range needed to facilitate factor integration, all selected spatial risk factors were used for field calculations in ArcMap 10.2. Fuzzy functions measure the degree of membership of data cells in a layer through control points that are set based on the relationship between the layer and disease/vectors on a scale of 0–1 (unsuitable to perfectly suitable). A linear relationship was applied to all factors, and the direction (increase/decrease) was defined for each factor, as shown in Supplementary 1. Temporal risk factors were standardized using a scale of 0–1 (low to high risk). For each temporal factor, the monthly raw data were divided by the maximum ownership value in each province (Supplementary 4 and 5). Standardization was performed using R released 4.0.0 [28].

### 2.6. Combination of All Factors

#### 2.6.1. Suitability Mapping

The WLC method was used to combine all the factors to generate suitability maps. This method produces a final weighted estimate of suitability for each location in the study area. In the WLC, each standardized factor was multiplied by its corresponding weight (Supplementary 1) and summed. Its equation is as:

(2) $$\begin{array}{c} S=\sum_{i=1}^{n}w_{i}v_{i}\left(a_{i}\right), \end{array}$$

where suitability is the overall value of risk factor at each subdistrict level, *n* is the number of risk factor, *w* is the weight of criteria *i* (*a*_*i*_), and *v* is the value functions of risk factor at layer *i* (*a*_*i*_). The WLC was implemented using ArcMap 10.2, to produce a final weighted estimate of suitability for each subdistrict in the study area in two steps. First, two output risk maps were produced based on two suitability maps for disease introduction into the targeted area and viral spread within the targeted area, including their consequences. Second, the final AI risk map was analyzed using a combination of two risk maps with the same weights. The outputs were presented as a vector map and choropleth map using quantiles as classification methods to illustrate the data.

### 2.7. Calculation of the Temporal Risk

To calculate the temporal risk in each province, all the factors were combined with their weights (Supplementary 1) using the following equation [29, 30]:

(3) $$\begin{array}{c} R=1-\left(1-S1\times W1\right)\times \ldots \times \left(1-S5\times W5\right), \end{array}$$

where *R* is monthly probability of risk for each poultry, *S*1–5 are the standardized values of risk factor 1–5, *W*1–5 are the weight of risk factor 1–5.

Subsequently, the probability of all provinces for each poultry type was averaged to calculate the risks at the country level. Calculations were performed using *R*, which released 4.0.0.

### 2.8. Map Validation

The suitability map was validated using the area under the curve (AUC) obtained from the receiver operating characteristic (ROC) analysis of the HPAI outbreak data for 2004–2008 in Thailand. The AUC values vary from 0.5 to 1.0. The ideal model had an AUC value of 1.0. [31].

## 3. Results

### 3.1. Spatial Risk

**Figure 1:**
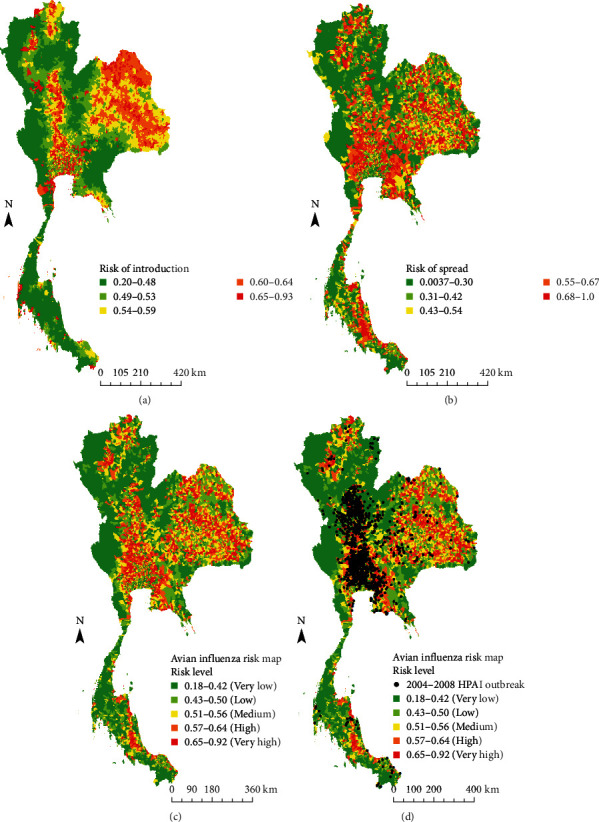
(a) Spatial risk map of avian influenza (AI) introduction, (b) spatial risk map of AI spread, (c) overall risk maps for AI, and (d) validation map based on data from reported outbreaks of highly pathogenic AI in Thailand during 2004–2008.

The AI risk maps of AI according to the possible introduction of the disease into poultry holdings, a spatial risk map of the virus spread throughout Thailand, and a final risk map of AI are shown in Figure 1a–c, respectively. At the subdistrict level, we found that high-risk areas were mainly located in the central and lower northern regions and some areas in the northeastern and southern regions. Moreover, 2045 HPAI outbreaks during 2004–2008 were used for map validation, as shown in Figure 1d. The results showed that the predictive capacity of the model had a moderate AUC value (AUC = 0.60, 95% CI = 0.58–0.61).

### 3.2. Temporal Risk

**Figure 2:**
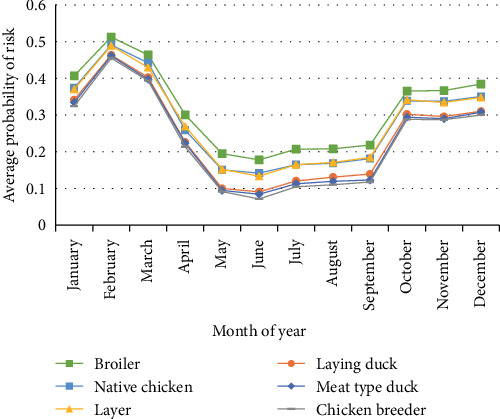
Average value of probability of risk in each province used to standardize the temporal risk factors, categorized by poultry subpopulation.

**Figure 3:**
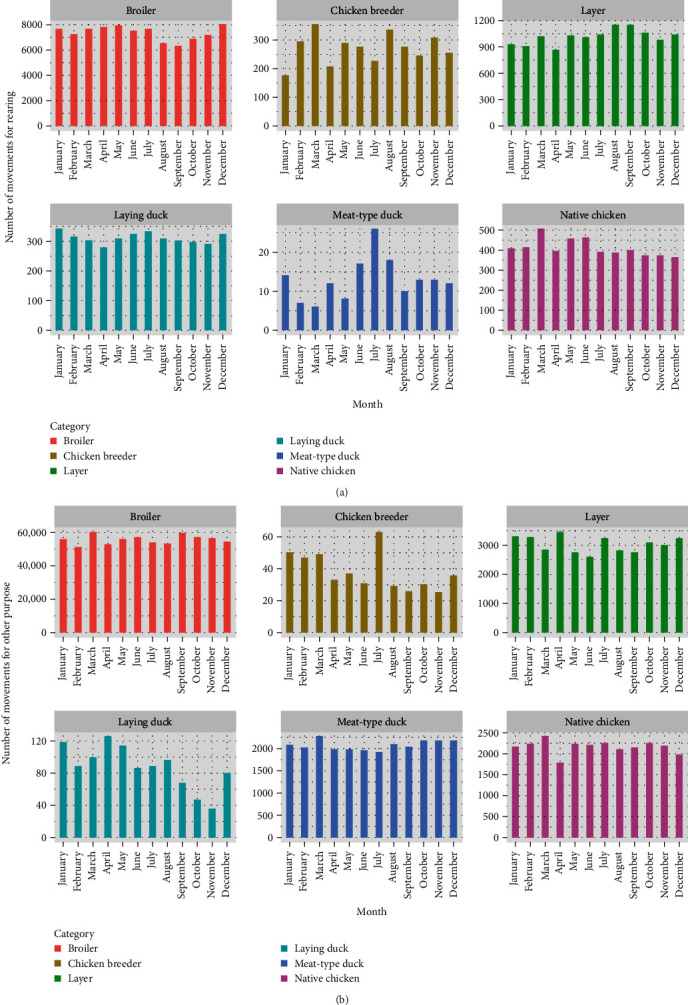
Temporal effect on the number of poultry movements (a) for rearing and (b) for other purposes.

As shown in Figure 2, the average probability of risk for all species was high from January to March and October to December. In addition, all the average probabilities of risk peaked in February (Figure 2). The results showed that broilers had the highest average probability of risk, followed by native chickens, layers, laying ducks, meat-type ducks, and chicken breeders. Considering the number of poultry movements shown in Figure 3, we found that the number of movements for each poultry species varied. In detail, poultry movement for rearing in Figure 3a showed that broilers, chicken breeders, layers, laying ducks, meat-type ducks, and native chickens peaked in December, March, August, September, January, July, and March, respectively. For other purposes, such as slaughter, sale, and export (Figure 3b), we found peaks of broiler, chicken breeder, layer, laying duck, meat-type duck, and native chicken in March, July, April, April, March, and March, respectively.

**Figure 4:**
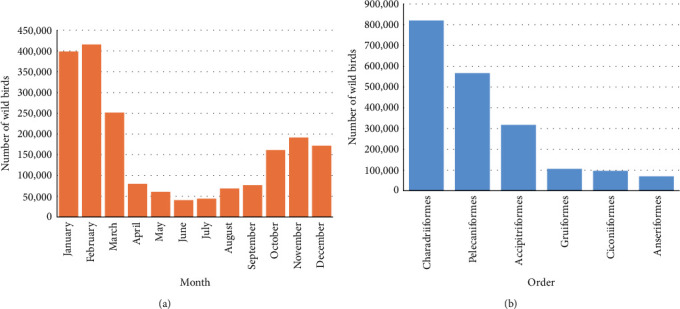
(a) Number of targeted wild birds observed each month and (b) number of targeted birds observed, categorized by order.

**Figure 5:**
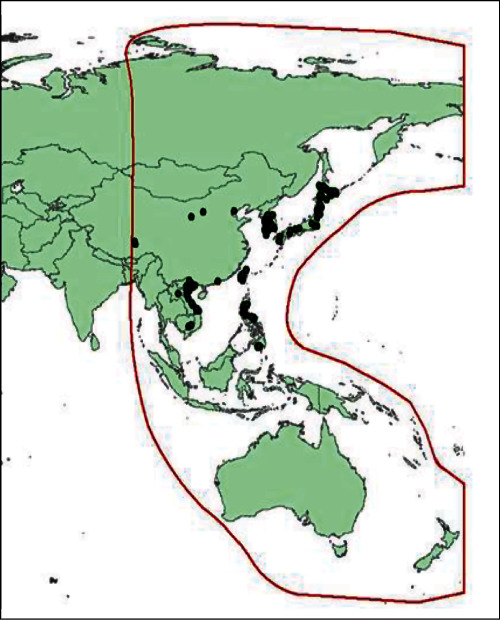
Highly pathogenic avian influenza outbreaks occurred between May 2021 and April 2022, in countries located in the East Asian–Australasian Flyway (EAAF).

**Figure 6:**
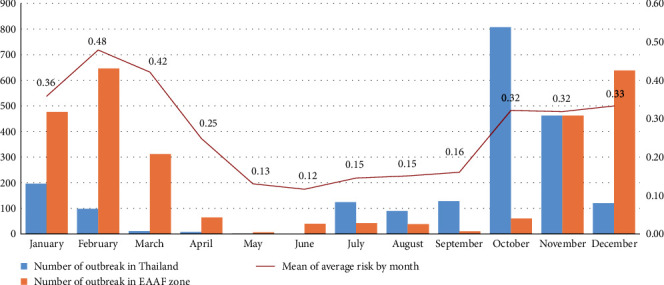
Possible temporal effects on avian influenza risk. Left *Y*-axis indicates the number of outbreaks used as scale for number of outbreaks in Thailand from 2004 to 2008 (Blue bar) and number of outbreaks in the East Asian–Australasian Flyway (EAAF) zone between May 2021 and April 2022 (Orange bar). Right *Y*-axis indicates mean average risk value (Red line) for all poultry at the provincial level in Thailand. *X*-axis represents time (in months).

For the targeted birds, the temporal effect was investigated based on the number of birds that appeared per month. We found that the number of observations for the targeted bird species was high in January and February (Figure 4a). The highest number of observations belonged to Charadriiformes, followed by Pelecaniformes, Accipitriformes, Gruiformes, Ciconiiformes, and Anseriformes (Figure 4b). Moreover, the spatial distribution of the HPAI in countries located in the EAAF was determined (Figure 5). We found that outbreaks of HPAI within the EAAF occurred most frequently in Southeast Asia and East Asia. Similarly, the number of HPAI outbreaks was high in February and December (Figure 6; orange bars). Thus, the seasonal pattern of risk for AI was correlated with the number of HPAI outbreaks in Thailand from 2004 to 2008 (Figure 6; blue bar), the number of HPAI outbreaks in the EAAF zone (Figure 6; orange bar), and the mean average poultry risk of AI by month at the provincial level (Figure 6; red line).

## 4. Discussion

This study investigates the possible spatial and temporal risks of AI in Thailand. Our analysis showed that high-risk areas were mainly located in the central and lower northern regions and some areas in the northeastern and southern regions of Thailand. We also found that the beginning and end of the year were temporal risks of AI in Thailand.

Our findings revealed that the areas suitable for HPAI introduction and spread were mainly in the central and lower northern regions of Thailand. This area is a hotspot that is strongly associated with proximity to water bodies, water bird density, and live poultry transportation. Most of this area consists of paddy fields that are used to cultivate rice for most of the year. Some wild birds select rice paddies as stopover sites during the migratory season, and free-grazing ducks and resident birds may be in indirect contact with contaminated water [3, 32]. Water samples from wild bird habitats have been found to have high AI virus subtype diversity [33]. The virus may remain infectious for up to 1 year when maintained in surface waters at ambient temperatures [34]. Consequently, landscapes shared between wild waterfowls and poultry are strongly associated with the occurrence of HPAI H5N1 [35]. Moreover, long-distance transportation of free-grazing ducks can lead to the spread of influenza virus [36]. Strengthening the biosecurity of poultry farms and testing for viruses before movement can reduce the risk of disease transmission.

In this study, the final risk map exhibited an AUC value of 0.65 when validated using the HPAI outbreak data from 2004 to 2008 in Thailand. An AUC value of 0.65 is considered moderate, indicating fair level accuracy in the model's predictive capabilities. This AUC value results from the study's comprehensive approach to AI risks, encompassing both LPAI and HPAI. Our analysis hypothesized that the worst-case scenario, in which ongoing surveillance systems are required, is more sensitive to both LPAI and HPAI. This would be more effective than an analysis specific only for HPAI. Moreover, in some situations, LPAI may mutate to HPAI. Therefore, a surveillance system based on our analysis is appropriate for the occurrence and spread of AI in future outbreaks. Nevertheless, our final risk map is very close to that of a previous study [3]. This indicates that the spatial risk is closely similar to previous outbreaks, on which relevant authorities need to be focused.

The results showed that the temporal effects were the beginning and the end of the year with consistency analysis from WOAH data, poultry movement data (move for rearing: broiler, chicken breeder, laying duck, and native chicken and move for other purposes: broiler, layer, meat-type duck, and native chicken), EAAF temporal risk, and the mean probability of average risk at the provincial level in Thailand. Moreover, seasonality analysis of global poultry HPAI outbreak in poultry between 2005 and 2016 showed that the spread began to rise in October and peaked in February [37]. This may be explained by experts identifying the number of observations of targeted bird species as an important risk factor. Migratory birds initially carried H5N1 from East Asia to breeding sites in Siberia via spring migration and global transmission to different flyways via autumn migration [38]. Thailand located in the EAAF zone which stretches from Russian far east and Alaska and southwards through East Asia and Southeast Asia [39]. Many bird species identified using the EAAF were confirmed to be HPAI-positive, which could be a risk pathway for introducing HPAI into domestic poultry in Thailand. Most waterbirds migrate from the northern temperate zone to Thailand during September–November and back during March–April [40, 41]. Wild birds belonging to Charadriiformes had the highest number of observations, including gulls, terns, plovers, shorebirds, and other species. The H5N1 virus currently causes high mortality in gulls, terns, and other seabird species across Europe [42]. These species, which migrate long distances, play a major role in the intercontinental spread of the virus through wild bird migrations [43, 44]. Previous studies have provided genomic data evidence demonstrating that HPAI can spread from Europe to Asia or from Asia to Europe through the migration of waterfowl [45]. Prevention of contracts between wild birds and poultry is essential for poultry farm biosecurity. In addition, the temporal risk analysis using data from the EAAF uses only HPAI because the data related to LPAI is incomplete. From our experience, we have found that many countries do not report cases of LPAI in the EAAF zone, necessitating the exclusion of LPAI data. This may result in underestimating the temporal risk when the analysis focuses on both HPAI and LPAI, especially if we consider only this specific aspect.

Several studies have used the MCDA approach to predict areas suitable for infectious diseases [46–49]. However, MCDA has a limitation—the subjectivity of experts' opinions in selecting risk factors, membership functions, and weights [21]. In our study, we relied on rich empirical data instead of expert opinions, resulting in more precise and less uncertain outcomes. We developed a method to evaluate temporal risk using MCDA techniques combined with expert opinions and empirical data, yielding more accurate results than conventional MCDA methods [22].

In this study, the eBird data we used were limited, covering only specific areas and the latest 5 years due to our computer's processing capacity. This led to some errors. However, we verified all results with our experts, who confirmed their precision. Experts also agreed that the spatial and temporal risks aligned with the current poultry farming situation in Thailand. Thus, these results can help design better surveillance systems. Another aspect to consider from our experience is the variation in risk factors estimated using linear models, which may deviate from reality more than nonlinear models. In this study, data derived from expert opinions often follow the linear model concept, as experts generally prefer linear models for their simplicity. This context might differ when using GIS-based MCDA with different experts. Typically, experts with knowledge in mathematical modeling or computational science tend to opt for more complex and nonlinear models. Therefore, this limitation should be taken into account.

In conclusion, this study introduces an enhanced GIS-based MCDA approach by integrating both spatial and temporal risk factors, representing a significant advancement over traditional methods that focus solely on spatial analysis. By leveraging rich empirical data instead of relying heavily on subjective expert opinions, we have reduced uncertainty and increased the precision of our predictions. The developed risk maps not only highlight high-risk areas for AI but also identify critical temporal windows for heightened surveillance efforts. These insights are crucial for designing and implementing more effective and timely surveillance systems, potentially leading to better disease prevention and control strategies. Our results align well with the current poultry farming situation in Thailand, providing actionable information for authorities to prioritize and allocate resources more efficiently.

## 5. Conclusions

This study describes the MCDA and risk assessment approaches that can be used to create risk maps and asses risk periods for AI in Thailand, using empirical data and expert opinions. These approaches can be employed in areas lacking disease events data; in addition, the method is easy to implement. The results of this study indicated that the risks posed by AI in Thailand were possibly caused by HPAI and/or LPAI. The risk maps prepared in this study revealed that the hotspot areas for HPAI were concentrated primarily in the central and lower northern regions of the country, which were relatively in close proximity to water bodies, water bird density, and live poultry transportation. The temporal risk period for AI was determined to be at the beginning and end of the year. These findings can assist veterinary services in improving risk-based surveillance programs and implementing other preventive and control strategies.

## Data Availability

The datasets from this study are available from the corresponding author upon reasonable request.

## Supporting Information

Supporting Information Supporting Information 1. Selected risk factors and methods used for the calculation, standardization, and weighting of avian influenza risk maps. Supporting Information 2. Nine-point continuous scale used for pairwise comparison of the factors. Supporting Information 3. Study framework for temporal and spatial risk assessment of Highly Pathogenic Avian influenza. Supporting Information 4. Saaty's random index (RI). Supporting Information 5. R code for temporal risk analysis.
