# Supplementary material for: Geospatial and Temporal Analysis of Avian Influenza Risk in Thailand: A GIS-Based Multi-Criteria Decision Analysis Approach for Enhanced Surveillance and Control
Source: Transbound Emerg Dis. 2024 Sep 13;2024:6474182. doi: 10.1155/2024/6474182 (PMC12017017; doi:10.1155/2024/6474182)
Supplement: Supporting Information — 1. Selected risk factors and methods used for the calculation, standardization, and weighting of avian influenza risk maps. Supporting Information 2. Nine-point continuous scale used for pairwise comparison of the factors. Supporting Information 3. Study framework for temporal and spatial risk assessment of Highly Pathogenic Avian influenza. Supporting Information 4. Saaty's random index (RI). Supporting Information 5. R code for temporal risk analysis. [file 6474182.f1.docx]

**Supplementary files**

**Supplementary 1.** Selected risk factors, and methods used for the calculation, standardization, and weighting of avian influenza risk maps.

| Risk | Factor | Unit | Standardization | Relationship | Within group weighing*^1^* | Between group weighing*^1^* | Source of data |
| --- | --- | --- | --- | --- | --- | --- | --- |
| Spatial risk for disease introduction in Thailand | Proximity to outbreak in neighboring country | km | 949 | decrease | 0.162 | 0.5 | DOPA*^2^* |
|  | Proximity to colonial waterbirds | km | 186 | decrease | 0.216 |  | DLD*^3^* |
|  | Proximity to water bodies | km | 152 | decrease | 0.259 |  | OCHA*^4^* |
|  | Water bird density | bird/km^2^ | 5 | increase | 0.236 |  | eBird*^5^* |
|  | Backyard poultry density | bird/km^2^ | 228 | increase | 0.069 |  | DLD |
|  | Free grazing duck density | bird/km^2^ | 43 | increase | 0.058 |  | DLD |
| Spatial risk for disease spread throughout Thailand | Live poultry movement | time | 9 | increase | 0.277 | 0.5 | DLD |
|  | Layer density | bird/km^2^ | 24 | increase | 0.183 |  | DLD |
|  | Broiler density | bird/km^2^ | 14 | increase | 0.039 |  | DLD |
|  | Farm duck density | bird/km^2^ | 74 | increase | 0.104 |  | DLD |
|  | Poultry holding density | farm/km^2^ | 13 | increase | 0.169 |  | DLD |
|  | Proximity to AI-ELISA positive farm | km | 759 | decrease | 0.229 |  | DLD |
| Temporal risk | Temporal effect on the number of poultry movements for rearing at provincial level | time/month | - | increase | 0.062 | - | DLD |
|  | Temporal effect on the number of poultry movements for other purposes at provincial level | time/month | - | increase | 0.128 |  | DLD |
|  | Observed number of targeted bird species | bird/month | - | increase | 0.301 |  | eBird |
|  | Number of outbreaks of AI in the countries within the East Asian–Australasian Flyway | time/month | - | increase | 0.330 |  | WOAH^6^ |
|  | Number of outbreaks of AI that occurred in Thailand | time/month | - | increase | 0.179 |  | DLD |

*^1^* Please note that the sum of within and between-group weighing must be equal to one. *^2^* DOPA = Department of Provincial Administration of Thailand. *^3^* DLD = Department of Livestock Development of Thailand. *^4^* OCHA = the United Nations Office for the Coordination of Humanitarian Affairs.*^5^* eBird = eBird dataset was obtained from https://ebird.org/home. *^6^* WOAH = World Organization for Animal Health.

**Supplementary 2.** Nine-point continuous scale used for pairwise comparison of the factors.

| Intensity of importance | Description |
| --- | --- |
| 1 | Equal importance |
| 3 | Moderate importance |
| 5 | Strong or essential importance |
| 7 | Very strong or demonstrated importance |
| 9 | Extreme importance |
| 2, 4, 6, 8 | Intermediate values |
| Reciprocals | Values for inverse comparison |


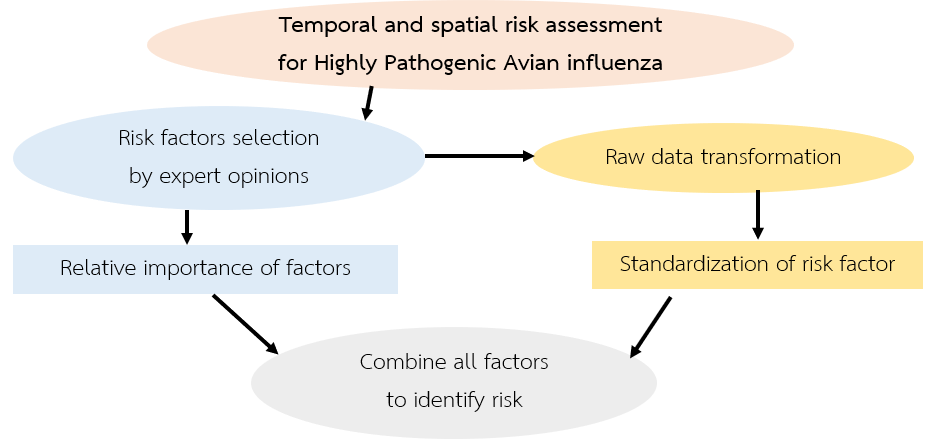


**Supplementary 3.** Study framework for temporal and spatial risk assessment of Highly Pathogenic Avian influenza.

**Supplementary 4.** Saaty’s random index (RI).

| No. of factors | 1 | 2 | 3 | 4 | 5 | 6 | 7 | 8 | 9 | 10 | 11 | 12 | 13 | 14 | 15 |
| --- | --- | --- | --- | --- | --- | --- | --- | --- | --- | --- | --- | --- | --- | --- | --- |
| RI | 0.00 | 0.00 | 0.58 | 0.90 | 1.12 | 1.24 | 1.32 | 1.41 | 1.46 | 1.49 | 1.51 | 1.54 | 1.56 | 1.57 | 1.58 |

**Supplementary 5.** R code for temporal risk analysis.

########Temporal R code############

########Code was updated and tested on 6 August 2024##########

rm()

#####1 Breeder

for(i in c(10:27,30:58,60:67,70:77,80:86,90:96)) {

MB <- read.table("D:/R/Temporal/Input/1TargetedBirds.csv", header = T, sep =",")

P1 <- MB[MB$Pco == i,c("Month","NUMBER")]

## P1 <- MB[MB$Pco,c("Month","COUNT")]

P1$Mcode <- ifelse(P1$Month == "January", 1,

ifelse(P1$Month == "February", 2,

ifelse(P1$Month == "March", 3,

ifelse(P1$Month == "April", 4,

ifelse(P1$Month == "May", 5,

ifelse(P1$Month == "June", 6,

ifelse(P1$Month == "July", 7,

ifelse(P1$Month == "August", 8,

ifelse(P1$Month == "September", 9,

ifelse(P1$Month == "October", 10,

ifelse(P1$Month == "November",11,12)))))))))))

P1 <- P1[,c("Mcode","NUMBER")]

names(P1) <- c("MONTH","MiBird")

####Movement Rearing

MR <- read.table("D:/R/Temporal/Input/3BreederRear.csv", header = T, sep =",")

P2 <- MR[MR$Pco == i,c("month","Count")]

## P2 <- LM[,c("month","move")]

names(P2) <- c("MONTH","MoveR")

MONTH1 <- as.data.frame(c(1:12))

names(MONTH1) <- "MONTH"

myVecMR <- match(MONTH1$MONTH,P2$MONTH)

MONTH1$MoveR <- P2$MoveR[myVecMR]

MONTH1$MoveR[is.na(MONTH1$MoveR)] <- 0

####Movement Rearing

MO <- read.table("D:/R/Temporal/Input/4breederOther.csv", header = T, sep =",")

P3 <- MO[MO$Pco == i,c("month","Count")]

## P2 <- LM[,c("month","move")]

names(P3) <- c("MONTH","MoveO")

MONTH2 <- as.data.frame(c(1:12))

names(MONTH2) <- "MONTH"

myVecMR <- match(MONTH2$MONTH,P3$MONTH)

MONTH2$MoveO <- P3$MoveO[myVecMR]

MONTH2$MoveO[is.na(MONTH2$MoveO)] <- 0

####Outbreak in flyway

OB <- read.table("D:/R/Temporal/Input/2outbreakdata.csv", header = T, sep =",")

P4 <- OB[OB$Pco == i,c("month","outbreak")]

## P3 <- LM[,c("month","move")]

names(P4) <- c("MONTH","outbreak")

####Outbreak in Thailand

OBinth <- read.table("D:/R/Temporal/Input/5outbreakinThailand.csv", header = T, sep =",")

P5 <- OBinth[OBinth$Pco == i,c("month","outbreak")]

## P3 <- LM[,c("month","move")]

names(P5) <- c("MONTH","OBTH")

#####Combine 3 variables by month

mySet <- P4

myV1 <- match(mySet$MONTH, P1$MONTH)

mySet$MiBird <- P1$MiBird[myV1]

myV2 <- match(mySet$MONTH, MONTH1$MONTH)

mySet$MoveR <- MONTH1$MoveR[myV2]

myV3 <- match(mySet$MONTH, MONTH2$MONTH)

mySet$MoveO <- MONTH2$MoveO[myV3]

myV4 <- match(mySet$MONTH, P5$MONTH)

mySet$OBTH <- P5$OBTH[myV4]

mySet[is.na(mySet)] <- 0

###Standardize variables

mySet$SDmoveR <- mySet$MoveR / max(mySet$MoveR)

mySet$SDmoveO <- mySet$MoveO / max(mySet$MoveO)

mySet$SDmig <- mySet$MiBird / max(mySet$MiBird)

mySet$SDoutbreak <- mySet$outbreak / max(mySet$outbreak)

mySet$SDOBTH <- mySet$OBTH / max(mySet$OBTH)

mySet[is.na(mySet)] <- 0

#mySet$SDpro[is.na(mySet$SDpro)] <- 1

###Set weight W1 = Movement, W2 = Migratory bird, W3 = Production

W1 <- 0.1560 #Movement to rearing

W2 <- 0.0933 #movement

W3 <- 0.4382 #targeted birds

W4 <- 0.2591#outbreak in flyway

W5 <- 0.0543 #outbreak in Thailand

mySet$SDmoveRW <- mySet$SDmoveR * W1

mySet$SDmoveOW <- mySet$SDmoveO *W2

mySet$SDmigW <- mySet$SDmig * W3

mySet$SDoutbreakW <- mySet$SDoutbreak * W4

mySet$SDOBTHW <- mySet$SDOBTH * W5

###Calculate probability

mySet$Prob <- 1-(1-mySet$SDmoveRW)*(1-mySet$SDmoveOW)*(1-mySet$SDmigW)*(1-mySet$SDoutbreakW)*(1-mySet$SDOBTHW)

###Save output by province

write.table(mySet, file=paste("D:/R/Temporal/Output/Breeder_PV_", i ,".csv",sep=""),dec=".",sep=",",row.names=F)

}

########## 2. Broiler

for(i in c(10:27,30:58,60:67,70:77,80:86,90:96)) {

MB <- read.table("D:/R/Temporal/Input/1TargetedBirds.csv", header = T, sep =",")

P1 <- MB[MB$Pco == i,c("Month","NUMBER")]

## P1 <- MB[MB$Pco,c("Month","COUNT")]

P1$Mcode <- ifelse(P1$Month == "January", 1,

ifelse(P1$Month == "February", 2,

ifelse(P1$Month == "March", 3,

ifelse(P1$Month == "April", 4,

ifelse(P1$Month == "May", 5,

ifelse(P1$Month == "June", 6,

ifelse(P1$Month == "July", 7,

ifelse(P1$Month == "August", 8,

ifelse(P1$Month == "September", 9,

ifelse(P1$Month == "October", 10,

ifelse(P1$Month == "November",11,12)))))))))))

P1 <- P1[,c("Mcode","NUMBER")]

names(P1) <- c("MONTH","MiBird")

####Movement Rearing

MR <- read.table("D:/R/Temporal/Input/BroilerRear.csv", header = T, sep =",")

P2 <- MR[MR$Pco == i,c("month","Count")]

## P2 <- LM[,c("month","move")]

names(P2) <- c("MONTH","MoveR")

MONTH1 <- as.data.frame(c(1:12))

names(MONTH1) <- "MONTH"

myVecMR <- match(MONTH1$MONTH,P2$MONTH)

MONTH1$MoveR <- P2$MoveR[myVecMR]

MONTH1$MoveR[is.na(MONTH1$MoveR)] <- 0

####Movement Rearing

MO <- read.table("D:/R/Temporal/Input/BroilerOther.csv", header = T, sep =",")

P3 <- MO[MO$Pco == i,c("month","Count")]

## P2 <- LM[,c("month","move")]

names(P3) <- c("MONTH","MoveO")

MONTH2 <- as.data.frame(c(1:12))

names(MONTH2) <- "MONTH"

myVecMR <- match(MONTH2$MONTH,P3$MONTH)

MONTH2$MoveO <- P3$MoveO[myVecMR]

MONTH2$MoveO[is.na(MONTH2$MoveO)] <- 0

####Outbreak

OB <- read.table("D:/R/Temporal/Input/2outbreakdata.csv", header = T, sep =",")

P4 <- OB[OB$Pco == i,c("month","outbreak")]

## P3 <- LM[,c("month","move")]

names(P4) <- c("MONTH","outbreak")

####Outbreak in Thailand

OBinth <- read.table("D:/R/Temporal/Input/5outbreakinThailand.csv", header = T, sep =",")

P5 <- OBinth[OBinth$Pco == i,c("month","outbreak")]

## P3 <- LM[,c("month","move")]

names(P5) <- c("MONTH","OBTH")

#####Combine 3 variables by month

mySet <- P4

myV1 <- match(mySet$MONTH, P1$MONTH)

mySet$MiBird <- P1$MiBird[myV1]

myV2 <- match(mySet$MONTH, MONTH1$MONTH)

mySet$MoveR <- MONTH1$MoveR[myV2]

myV3 <- match(mySet$MONTH, MONTH2$MONTH)

mySet$MoveO <- MONTH2$MoveO[myV3]

myV4 <- match(mySet$MONTH, P5$MONTH)

mySet$OBTH <- P5$OBTH[myV4]

mySet[is.na(mySet)] <- 0

###Standardize variables

mySet$SDmoveR <- mySet$MoveR / max(mySet$MoveR)

mySet$SDmoveO <- mySet$MoveO / max(mySet$MoveO)

mySet$SDmig <- mySet$MiBird / max(mySet$MiBird)

mySet$SDoutbreak <- mySet$outbreak / max(mySet$outbreak)

mySet$SDOBTH <- mySet$OBTH / max(mySet$OBTH)

mySet[is.na(mySet)] <- 0

#mySet$SDpro[is.na(mySet$SDpro)] <- 1

###Set weight W1 = Movement, W2 = Migratory bird, W3 = Production

W1 <- 0.1560 #Movement to rearing

W2 <- 0.0933 #movement

W3 <- 0.4382 #targeted birds

W4 <- 0.2591#outbreak in flyway

W5 <- 0.0543 #outbreak in Thailand

mySet$SDmoveRW <- mySet$SDmoveR * W1

mySet$SDmoveOW <- mySet$SDmoveO *W2

mySet$SDmigW <- mySet$SDmig * W3

mySet$SDoutbreakW <- mySet$SDoutbreak * W4

mySet$SDOBTHW <- mySet$SDOBTH * W5

###Calculate probability

mySet$Prob <- 1-(1-mySet$SDmoveRW)*(1-mySet$SDmoveOW)*(1-mySet$SDmigW)*(1-mySet$SDoutbreakW)*(1-mySet$SDOBTHW)

###Save output by province

write.table(mySet, file=paste("D:/R/Temporal/Output/Broiler_PV_", i ,".csv",sep=""),dec=".",sep=",",row.names=F)

}

######### 3. Laying duck

for(i in c(10:27,30:58,60:67,70:77,80:86,90:96)) {

MB <- read.table("D:/R/Temporal/Input/1TargetedBirds.csv", header = T, sep =",")

P1 <- MB[MB$Pco == i,c("Month","NUMBER")]

## P1 <- MB[MB$Pco,c("Month","COUNT")]

P1$Mcode <- ifelse(P1$Month == "January", 1,

ifelse(P1$Month == "February", 2,

ifelse(P1$Month == "March", 3,

ifelse(P1$Month == "April", 4,

ifelse(P1$Month == "May", 5,

ifelse(P1$Month == "June", 6,

ifelse(P1$Month == "July", 7,

ifelse(P1$Month == "August", 8,

ifelse(P1$Month == "September", 9,

ifelse(P1$Month == "October", 10,

ifelse(P1$Month == "November",11,12)))))))))))

P1 <- P1[,c("Mcode","NUMBER")]

names(P1) <- c("MONTH","MiBird")

####Movement Rearing

MR <- read.table("D:/R/Temporal/Input/LayDuckRear.csv", header = T, sep =",")

P2 <- MR[MR$Pco == i,c("month","Count")]

## P2 <- LM[,c("month","move")]

names(P2) <- c("MONTH","MoveR")

MONTH1 <- as.data.frame(c(1:12))

names(MONTH1) <- "MONTH"

myVecMR <- match(MONTH1$MONTH,P2$MONTH)

MONTH1$MoveR <- P2$MoveR[myVecMR]

MONTH1$MoveR[is.na(MONTH1$MoveR)] <- 0

####Movement Other

MO <- read.table("D:/R/Temporal/Input/LayDuckOther.csv", header = T, sep =",")

P3 <- MO[MO$Pco == i,c("month","Count")]

## P2 <- LM[,c("month","move")]

names(P3) <- c("MONTH","MoveO")

MONTH2 <- as.data.frame(c(1:12))

names(MONTH2) <- "MONTH"

myVecMR <- match(MONTH2$MONTH,P3$MONTH)

MONTH2$MoveO <- P3$MoveO[myVecMR]

MONTH2$MoveO[is.na(MONTH2$MoveO)] <- 0

####Outbreak

OB <- read.table("D:/R/Temporal/Input/2outbreakdata.csv", header = T, sep =",")

P4 <- OB[OB$Pco == i,c("month","outbreak")]

## P3 <- LM[,c("month","move")]

names(P4) <- c("MONTH","outbreak")

####Outbreak in Thailand

OBinth <- read.table("D:/R/Temporal/Input/5outbreakinThailand.csv", header = T, sep =",")

P5 <- OBinth[OBinth$Pco == i,c("month","outbreak")]

## P3 <- LM[,c("month","move")]

names(P5) <- c("MONTH","OBTH")

#####Combine 3 variables by month

mySet <- P4

myV1 <- match(mySet$MONTH, P1$MONTH)

mySet$MiBird <- P1$MiBird[myV1]

myV2 <- match(mySet$MONTH, MONTH1$MONTH)

mySet$MoveR <- MONTH1$MoveR[myV2]

myV3 <- match(mySet$MONTH, MONTH2$MONTH)

mySet$MoveO <- MONTH2$MoveO[myV3]

myV4 <- match(mySet$MONTH, P5$MONTH)

mySet$OBTH <- P5$OBTH[myV4]

mySet[is.na(mySet)] <- 0

###Standardize variables

mySet$SDmoveR <- mySet$MoveR / max(mySet$MoveR)

mySet$SDmoveO <- mySet$MoveO / max(mySet$MoveO)

mySet$SDmig <- mySet$MiBird / max(mySet$MiBird)

mySet$SDoutbreak <- mySet$outbreak / max(mySet$outbreak)

mySet$SDOBTH <- mySet$OBTH / max(mySet$OBTH)

mySet[is.na(mySet)] <- 0

#mySet$SDpro[is.na(mySet$SDpro)] <- 1

###Set weight W1 = Movement, W2 = Migratory bird, W3 = Production

W1 <- 0.1560 #Movement to rearing

W2 <- 0.0933 #movement

W3 <- 0.4382 #targeted birds

W4 <- 0.2591#outbreak in flyway

W5 <- 0.0543 #outbreak in Thailand

mySet$SDmoveRW <- mySet$SDmoveR * W1

mySet$SDmoveOW <- mySet$SDmoveO *W2

mySet$SDmigW <- mySet$SDmig * W3

mySet$SDoutbreakW <- mySet$SDoutbreak * W4

mySet$SDOBTHW <- mySet$SDOBTH * W5

###Calculate probability

mySet$Prob <- 1-(1-mySet$SDmoveRW)*(1-mySet$SDmoveOW)*(1-mySet$SDmigW)*(1-mySet$SDoutbreakW)*(1-mySet$SDOBTHW)

###Save output by province

write.table(mySet, file=paste("D:/R/Temporal/Output/LayingDuck_PV_", i ,".csv",sep=""),dec=".",sep=",",row.names=F)

}

##### 4. Layer

for(i in c(10:27,30:58,60:67,70:77,80:86,90:96)) {

MB <- read.table("D:/R/Temporal/Input/1TargetedBirds.csv", header = T, sep =",")

P1 <- MB[MB$Pco == i,c("Month","NUMBER")]

## P1 <- MB[MB$Pco,c("Month","COUNT")]

P1$Mcode <- ifelse(P1$Month == "January", 1,

ifelse(P1$Month == "February", 2,

ifelse(P1$Month == "March", 3,

ifelse(P1$Month == "April", 4,

ifelse(P1$Month == "May", 5,

ifelse(P1$Month == "June", 6,

ifelse(P1$Month == "July", 7,

ifelse(P1$Month == "August", 8,

ifelse(P1$Month == "September", 9,

ifelse(P1$Month == "October", 10,

ifelse(P1$Month == "November",11,12)))))))))))

P1 <- P1[,c("Mcode","NUMBER")]

names(P1) <- c("MONTH","MiBird")

####Movement Rearing

MR <- read.table("D:/R/Temporal/Input/LayerRear.csv", header = T, sep =",")

P2 <- MR[MR$Pco == i,c("month","Count")]

## P2 <- LM[,c("month","move")]

names(P2) <- c("MONTH","MoveR")

MONTH1 <- as.data.frame(c(1:12))

names(MONTH1) <- "MONTH"

myVecMR <- match(MONTH1$MONTH,P2$MONTH)

MONTH1$MoveR <- P2$MoveR[myVecMR]

MONTH1$MoveR[is.na(MONTH1$MoveR)] <- 0

####Movement Other

MO <- read.table("D:/R/Temporal/Input/LayerOther.csv", header = T, sep =",")

P3 <- MO[MO$Pco == i,c("month","Count")]

## P2 <- LM[,c("month","move")]

names(P3) <- c("MONTH","MoveO")

MONTH2 <- as.data.frame(c(1:12))

names(MONTH2) <- "MONTH"

myVecMR <- match(MONTH2$MONTH,P3$MONTH)

MONTH2$MoveO <- P3$MoveO[myVecMR]

MONTH2$MoveO[is.na(MONTH2$MoveO)] <- 0

####Outbreak

OB <- read.table("D:/R/Temporal/Input/2outbreakdata.csv", header = T, sep =",")

P4 <- OB[OB$Pco == i,c("month","outbreak")]

## P3 <- LM[,c("month","move")]

names(P4) <- c("MONTH","outbreak")

####Outbreak in Thailand

OBinth <- read.table("D:/R/Temporal/Input/5outbreakinThailand.csv", header = T, sep =",")

P5 <- OBinth[OBinth$Pco == i,c("month","outbreak")]

## P3 <- LM[,c("month","move")]

names(P5) <- c("MONTH","OBTH")

#####Combine 3 variables by month

mySet <- P4

myV1 <- match(mySet$MONTH, P1$MONTH)

mySet$MiBird <- P1$MiBird[myV1]

myV2 <- match(mySet$MONTH, MONTH1$MONTH)

mySet$MoveR <- MONTH1$MoveR[myV2]

myV3 <- match(mySet$MONTH, MONTH2$MONTH)

mySet$MoveO <- MONTH2$MoveO[myV3]

myV4 <- match(mySet$MONTH, P5$MONTH)

mySet$OBTH <- P5$OBTH[myV4]

mySet[is.na(mySet)] <- 0

###Standardize variables

mySet$SDmoveR <- mySet$MoveR / max(mySet$MoveR)

mySet$SDmoveO <- mySet$MoveO / max(mySet$MoveO)

mySet$SDmig <- mySet$MiBird / max(mySet$MiBird)

mySet$SDoutbreak <- mySet$outbreak / max(mySet$outbreak)

mySet$SDOBTH <- mySet$OBTH / max(mySet$OBTH)

mySet[is.na(mySet)] <- 0

#mySet$SDpro[is.na(mySet$SDpro)] <- 1

###Set weight W1 = Movement, W2 = Migratory bird, W3 = Production

W1 <- 0.1560 #Movement to rearing

W2 <- 0.0933 #movement

W3 <- 0.4382 #targeted birds

W4 <- 0.2591#outbreak in flyway

W5 <- 0.0543 #outbreak in Thailand

mySet$SDmoveRW <- mySet$SDmoveR * W1

mySet$SDmoveOW <- mySet$SDmoveO *W2

mySet$SDmigW <- mySet$SDmig * W3

mySet$SDoutbreakW <- mySet$SDoutbreak * W4

mySet$SDOBTHW <- mySet$SDOBTH * W5

###Calculate probability

mySet$Prob <- 1-(1-mySet$SDmoveRW)*(1-mySet$SDmoveOW)*(1-mySet$SDmigW)*(1-mySet$SDoutbreakW)*(1-mySet$SDOBTHW)

###Save output by province

write.table(mySet, file=paste("D:/R/Temporal/Output/Layer_PV_", i ,".csv",sep=""),dec=".",sep=",",row.names=F)

}

#### 5. Meat duck

for(i in c(10:27,30:58,60:67,70:77,80:86,90:96)) {

MB <- read.table("D:/R/Temporal/Input/1TargetedBirds.csv", header = T, sep =",")

P1 <- MB[MB$Pco == i,c("Month","NUMBER")]

## P1 <- MB[MB$Pco,c("Month","COUNT")]

P1$Mcode <- ifelse(P1$Month == "January", 1,

ifelse(P1$Month == "February", 2,

ifelse(P1$Month == "March", 3,

ifelse(P1$Month == "April", 4,

ifelse(P1$Month == "May", 5,

ifelse(P1$Month == "June", 6,

ifelse(P1$Month == "July", 7,

ifelse(P1$Month == "August", 8,

ifelse(P1$Month == "September", 9,

ifelse(P1$Month == "October", 10,

ifelse(P1$Month == "November",11,12)))))))))))

P1 <- P1[,c("Mcode","NUMBER")]

names(P1) <- c("MONTH","MiBird")

####Movement Rearing

MR <- read.table("D:/R/Temporal/Input/MeatDuckRear.csv", header = T, sep =",")

P2 <- MR[MR$Pco == i,c("month","Count")]

## P2 <- LM[,c("month","move")]

names(P2) <- c("MONTH","MoveR")

MONTH1 <- as.data.frame(c(1:12))

names(MONTH1) <- "MONTH"

myVecMR <- match(MONTH1$MONTH,P2$MONTH)

MONTH1$MoveR <- P2$MoveR[myVecMR]

MONTH1$MoveR[is.na(MONTH1$MoveR)] <- 0

####Movement Other

MO <- read.table("D:/R/Temporal/Input/MeatduckOther.csv", header = T, sep =",")

P3 <- MO[MO$Pco == i,c("month","Count")]

## P2 <- LM[,c("month","move")]

names(P3) <- c("MONTH","MoveO")

MONTH2 <- as.data.frame(c(1:12))

names(MONTH2) <- "MONTH"

myVecMR <- match(MONTH2$MONTH,P3$MONTH)

MONTH2$MoveO <- P3$MoveO[myVecMR]

MONTH2$MoveO[is.na(MONTH2$MoveO)] <- 0

####Outbreak

OB <- read.table("D:/R/Temporal/Input/2outbreakdata.csv", header = T, sep =",")

P4 <- OB[OB$Pco == i,c("month","outbreak")]

## P3 <- LM[,c("month","move")]

names(P4) <- c("MONTH","outbreak")

####Outbreak in Thailand

OBinth <- read.table("D:/R/Temporal/Input/5outbreakinThailand.csv", header = T, sep =",")

P5 <- OBinth[OBinth$Pco == i,c("month","outbreak")]

## P3 <- LM[,c("month","move")]

names(P5) <- c("MONTH","OBTH")

#####Combine 3 variables by month

mySet <- P4

myV1 <- match(mySet$MONTH, P1$MONTH)

mySet$MiBird <- P1$MiBird[myV1]

myV2 <- match(mySet$MONTH, MONTH1$MONTH)

mySet$MoveR <- MONTH1$MoveR[myV2]

myV3 <- match(mySet$MONTH, MONTH2$MONTH)

mySet$MoveO <- MONTH2$MoveO[myV3]

myV4 <- match(mySet$MONTH, P5$MONTH)

mySet$OBTH <- P5$OBTH[myV4]

mySet[is.na(mySet)] <- 0

###Standardize variables

mySet$SDmoveR <- mySet$MoveR / max(mySet$MoveR)

mySet$SDmoveO <- mySet$MoveO / max(mySet$MoveO)

mySet$SDmig <- mySet$MiBird / max(mySet$MiBird)

mySet$SDoutbreak <- mySet$outbreak / max(mySet$outbreak)

mySet$SDOBTH <- mySet$OBTH / max(mySet$OBTH)

mySet[is.na(mySet)] <- 0

#mySet$SDpro[is.na(mySet$SDpro)] <- 1

###Set weight W1 = Movement, W2 = Migratory bird, W3 = Production

W1 <- 0.1560 #Movement to rearing

W2 <- 0.0933 #movement

W3 <- 0.4382 #targeted birds

W4 <- 0.2591#outbreak in flyway

W5 <- 0.0543 #outbreak in Thailand

mySet$SDmoveRW <- mySet$SDmoveR * W1

mySet$SDmoveOW <- mySet$SDmoveO *W2

mySet$SDmigW <- mySet$SDmig * W3

mySet$SDoutbreakW <- mySet$SDoutbreak * W4

mySet$SDOBTHW <- mySet$SDOBTH * W5

###Calculate probability

mySet$Prob <- 1-(1-mySet$SDmoveRW)*(1-mySet$SDmoveOW)*(1-mySet$SDmigW)*(1-mySet$SDoutbreakW)*(1-mySet$SDOBTHW)

###Save output by province

write.table(mySet, file=paste("D:/R/Temporal/Output/MeatDuck_PV_", i ,".csv",sep=""),dec=".",sep=",",row.names=F)

}

##### 6. Native chicken

for(i in c(10:27,30:58,60:67,70:77,80:86,90:96)) {

MB <- read.table("D:/R/Temporal/Input/1TargetedBirds.csv", header = T, sep =",")

P1 <- MB[MB$Pco == i,c("Month","NUMBER")]

## P1 <- MB[MB$Pco,c("Month","COUNT")]

P1$Mcode <- ifelse(P1$Month == "January", 1,

ifelse(P1$Month == "February", 2,

ifelse(P1$Month == "March", 3,

ifelse(P1$Month == "April", 4,

ifelse(P1$Month == "May", 5,

ifelse(P1$Month == "June", 6,

ifelse(P1$Month == "July", 7,

ifelse(P1$Month == "August", 8,

ifelse(P1$Month == "September", 9,

ifelse(P1$Month == "October", 10,

ifelse(P1$Month == "November",11,12)))))))))))

P1 <- P1[,c("Mcode","NUMBER")]

names(P1) <- c("MONTH","MiBird")

####Movement Rearing

MR <- read.table("D:/R/Temporal/Input/NativeRear.csv", header = T, sep =",")

P2 <- MR[MR$Pco == i,c("month","Count")]

## P2 <- LM[,c("month","move")]

names(P2) <- c("MONTH","MoveR")

MONTH1 <- as.data.frame(c(1:12))

names(MONTH1) <- "MONTH"

myVecMR <- match(MONTH1$MONTH,P2$MONTH)

MONTH1$MoveR <- P2$MoveR[myVecMR]

MONTH1$MoveR[is.na(MONTH1$MoveR)] <- 0

####Movement Other

MO <- read.table("D:/R/Temporal/Input/NativeOther.csv", header = T, sep =",")

P3 <- MO[MO$Pco == i,c("month","Count")]

## P2 <- LM[,c("month","move")]

names(P3) <- c("MONTH","MoveO")

MONTH2 <- as.data.frame(c(1:12))

names(MONTH2) <- "MONTH"

myVecMR <- match(MONTH2$MONTH,P3$MONTH)

MONTH2$MoveO <- P3$MoveO[myVecMR]

MONTH2$MoveO[is.na(MONTH2$MoveO)] <- 0

####Outbreak

OB <- read.table("D:/R/Temporal/Input/2outbreakdata.csv", header = T, sep =",")

P4 <- OB[OB$Pco == i,c("month","outbreak")]

## P3 <- LM[,c("month","move")]

names(P4) <- c("MONTH","outbreak")

####Outbreak in Thailand

OBinth <- read.table("D:/R/Temporal/Input/5outbreakinThailand.csv", header = T, sep =",")

P5 <- OBinth[OBinth$Pco == i,c("month","outbreak")]

## P3 <- LM[,c("month","move")]

names(P5) <- c("MONTH","OBTH")

#####Combine 3 variables by month

mySet <- P4

myV1 <- match(mySet$MONTH, P1$MONTH)

mySet$MiBird <- P1$MiBird[myV1]

myV2 <- match(mySet$MONTH, MONTH1$MONTH)

mySet$MoveR <- MONTH1$MoveR[myV2]

myV3 <- match(mySet$MONTH, MONTH2$MONTH)

mySet$MoveO <- MONTH2$MoveO[myV3]

myV4 <- match(mySet$MONTH, P5$MONTH)

mySet$OBTH <- P5$OBTH[myV4]

mySet[is.na(mySet)] <- 0

###Standardize variables

mySet$SDmoveR <- mySet$MoveR / max(mySet$MoveR)

mySet$SDmoveO <- mySet$MoveO / max(mySet$MoveO)

mySet$SDmig <- mySet$MiBird / max(mySet$MiBird)

mySet$SDoutbreak <- mySet$outbreak / max(mySet$outbreak)

mySet$SDOBTH <- mySet$OBTH / max(mySet$OBTH)

mySet[is.na(mySet)] <- 0

#mySet$SDpro[is.na(mySet$SDpro)] <- 1

###Set weight W1 = Movement, W2 = Migratory bird, W3 = Production

W1 <- 0.1560 #Movement to rearing

W2 <- 0.0933 #movement

W3 <- 0.4382 #targeted birds

W4 <- 0.2591#outbreak in flyway

W5 <- 0.0543 #outbreak in Thailand

mySet$SDmoveRW <- mySet$SDmoveR * W1

mySet$SDmoveOW <- mySet$SDmoveO *W2

mySet$SDmigW <- mySet$SDmig * W3

mySet$SDoutbreakW <- mySet$SDoutbreak * W4

mySet$SDOBTHW <- mySet$SDOBTH * W5

###Calculate probability

mySet$Prob <- 1-(1-mySet$SDmoveRW)*(1-mySet$SDmoveOW)*(1-mySet$SDmigW)*(1-mySet$SDoutbreakW)*(1-mySet$SDOBTHW)

###Save output by province

write.table(mySet, file=paste("D:/R/Temporal/Output/Native_PV_", i ,".csv",sep=""),dec=".",sep=",",row.names=F)

}
